# Efficacy of acupuncture in improving the symptoms and the quality of life of patients with moderate or severe acne vulgaris: study protocol for a randomized controlled trial

**DOI:** 10.1186/s13063-020-04346-7

**Published:** 2020-06-23

**Authors:** Ruimin Jiao, Man Huang, Weina Zhang, Zhishun Liu

**Affiliations:** 1 Department of Acupuncture, Guang’anmen Hospital, China Academy of Chinese Medical Sciences, No. 5 Beixiange Street, Xicheng District, Beijing, China; 2 China Academy of Chinese Medical Sciences, Beijing, China

**Keywords:** Acupuncture, Acne vulgaris, Quality of life, Randomized controlled trial, Protocol

## Abstract

**Background:**

Acne vulgaris (AV) is a common chronic dermatologic disease that tends to impair the appearance and quality of life (QoL) of patients. Although several trials have indicated the effectiveness of acupuncture for treatment of patients with AV, the results of these trials have been contested, owing to potential bias in their design. Thus, there is a lack of robust data to evaluate the efficacy of acupuncture in patients with AV. In addition, none of the previous clinical trials of acupuncture therapy for AV used QoL as a primary outcome or employed a sham acupuncture control arm. The aim of the present study is to evaluate the effectiveness of acupuncture in treating the symptoms and QoL of patients with moderate or severe AV.

**Methods/design:**

One hundred eligible participants with AV will be randomly assigned to an acupuncture or a sham acupuncture group (1:1 allocation). All participants will receive 4-week treatment comprising a total of 12 sessions (3 sessions per week). The primary outcome will be change from baseline in the Skindex-16 scale total score at treatment completion. The secondary outcomes will be Skindex-16 subscale score, Dermatology Life Quality Index scale total score, total lesion count and inflammatory lesion count, visual analogue scale scores for assessment of itch and pain, patient expectations of acupuncture, and blinding of the effect of sham acupuncture. Follow-up evaluation will be performed at weeks 16 and 28. All outcome analyses will be performed in the intention-to-treat population.

**Discussion:**

We expect to evaluate the effectiveness of acupuncture in ameliorating the symptoms and improving the QoL of patients with moderate or severe AV compared with sham acupuncture with more robust evidence. The limitations of the trial design are its single-center scope, relatively small sample size, and lack of blinding of the acupuncturists.

**Trial registration:**

Chinese Clinical Trial Registry, ChiCTR-1900023649. Registered on January 2, 2019.

## Background

Acne vulgaris (AV) is a common chronic dermatologic disease involving pilosebaceous units [1, 2]. Skin lesions are the main symptoms of AV; they typically appear over the face, chest, and back [1, 3]. An estimated 79–95% of the adolescent population is affected by AV [4]. The skin lesions include comedones, erythematous papules, pustules, nodules, deep pustules, and scarring. The occurrence of AV lesions on the face may induce anxiety and decrease self-esteem [5, 6], leading to a decline in the quality of life (QoL) [5–8].

The American Society of Dermatology recommends benzoyl peroxide, topical retinoids, or systemic antibiotic therapy as the first-line treatment for mild to severe AV [1–3]. However, these pharmaceutical therapies may cause side effects of drying, peeling, erythema, and skin irritation; in addition, prolonged treatment may result in drug resistance, and the associated recurrence rate is relatively high [9]. Therefore, there is an increasing interest in exploring natural and safe therapies for AV [9, 10]. These natural and safer treatment options include complementary and alternative treatment remedies, such as herbal medicine and acupuncture [9, 10].

Several trials have indicated that acupuncture may alleviate the skin lesions and improve the QoL of patients with AV [11–15]. However, these trials were affected by potential bias due to small sample sizes, nonplacebo/sham/waiting list control, or use of self-defined outcome measures; thus, they did not provide robust evidence of the efficacy of acupuncture in patients with AV. Therefore, we conducted a pilot trial (unpublished) from April 2017 to March 2018 in which 42 patients with moderate or severe AV were randomly allocated to an acupuncture group (*n* = 21) or a sham acupuncture group (*n* = 21). After a 4-week treatment, the number of inflammatory lesions in the acupuncture group (*n* = 21) was 35.62 ± 15.51, with a reduction of 6.62 ± 15.42 from baseline. The number of inflammatory lesions in the sham acupuncture group (*n* = 21) was 30.05 ± 21.16, with a reduction of 15.10 ± 20.13 from the baseline. The difference between the two groups was 8.48 ± 7.74, and there was no significant between-group difference with the *p* = 0.137 due to *p* > 0.05 was considered nonsignificant. These findings contradict the results of previous trials [11–14], and authors of a systematic review of AV also reported that there was no difference between acupuncture and the waiting list for reducing the number of inflammatory lesions [16]. Unlike drugs, which act directly on the pathogen, acupuncture restores normal functions through motivating or inducing the inherent regulatory system [17]. Therefore, acupuncture may relieve other symptoms of AV (itch or pain) [18, 19] and improve the QoL [11]. The Skindex-16 scale is a sensitive and specific tool for assessing the symptoms and QoL of patients with AV, and it includes seven questions related to the symptoms (itch or pain) and characteristics (skin lesions) of AV [20, 21]. Following the 4-week treatment, the total score on the Skindex-16 scale for acupuncture (*n* = 21) was 19.89 ± 15.40, with a reduction of 12.50 ± 19.09 from the baseline. The total score on the Skindex-16 scale for sham acupuncture (*n* = 21) was 30.43 ± 19.39, with a reduction of 0.40 ± 21.12 from the baseline. The difference between the two groups was − 12.9 ± 11, and there was a significant between-group difference in this instance with the *p* = 0.044 due to *p* < 0.05 was considered statistically significant. Therefore, the results of our pilot study indicated that acupuncture may help to relieve total symptoms and improve the QoL of patients with moderate or severe AV. So far, there are two systematic reviews that recommend using the measure of QoL for evaluating the effect of acupuncture on AV [10, 16], but no clinical trials have been conducted that use the measure of QoL to evaluate the effect of acupuncture on the symptoms and the QoL of patients with AV. Therefore, we intend to use the Skindex-16 scale as a primary outcome measure in our planned study to evaluate the effect of acupuncture compared with sham acupuncture on the symptoms and QoL of patients with moderate or severe AV.

## Methods/design

### Study design

The proposed study is a prospective, randomized, sham acupuncture controlled trial with two parallel arms using a 1:1 allocation ratio. The trial will be conducted at the Guang’anmen Hospital, China Academy of Chinese Medical Sciences. The study protocol conforms to the Standard Protocol Items: Recommendations for Interventional Trials (SPIRIT) [22] and the Standards for Reporting Interventions in Clinical Trials of Acupuncture (STRICTA) [23].

### Study recruitment

Participants will be recruited between August 2019 and December 2020 at the Guang’ anmen Hospital via advertisements on posters, the WeChat app, and hospital websites. A dermatologist will be responsible for the screening, which will include diagnosis of the type of acne of patients and a series of physical examinations (discrimination between inflammatory and noninflammatory lesions of AV by the pictures of participants’ faces obtained using a digital camera) according to the diagnostic criteria for AV [2, 24] and the classification of lesions of AV [25], respectively.

All participants will be asked by a research assistant to read the informed consent form that describes the trial, randomization process for treatments, and the potential benefits and risks of the trial. Participants will be informed that both acupuncture and sham acupuncture may be effective for AV, and they will be randomly assigned to the acupuncture group or the sham acupuncture group. Participants will be allowed to withdraw from the trial at any time. Written informed consent will be obtained from all subjects prior to their enrollment.

The demographic and clinical characteristics of the participants at baseline will be recorded by a research assistant: age, sex, race, body mass index (in kg/m^2^), marital status, education level (primary education or below, secondary education, and tertiary education), time of sports activity (0–3 times/month, 1–2 times/week, 3–4 times/week, and ≥ 5 times/week), daily sleep duration (< 7 h, 7–9 h, and > 9 h), smoking history, history of alcohol intake, AV duration in months, and severity of AV as assessed by Global Acne Grading System (GAGS) score (none [0 score], mild [1–18 score], moderate [19–30 score], severe [31–38 score], and markedly severe [> 39 score]) [26].

### Randomization and allocation concealment

The randomization scheme has been prepared by the National Clinical Drug Testing Institute of the Guang’anmen Hospital. Participants will be randomly assigned to receive acupuncture or sham acupuncture treatment in a 1:1 ratio with the fixed block of 4. Sealed opaque envelopes will be used to ensure randomization concealment. The number of the randomization sequence and information on group allocation will be sealed in ordered envelopes. With the inclusion of patients, those envelopes will be opened one by one in sequence. The envelopes will be kept by a research assistant who is not involved in the treatment or assessment. Additionally, assistant researchers will perform a baseline assessment of all participants (− 1 week to 0 week) prior to randomization.

### Blinding

**Fig. 1:**
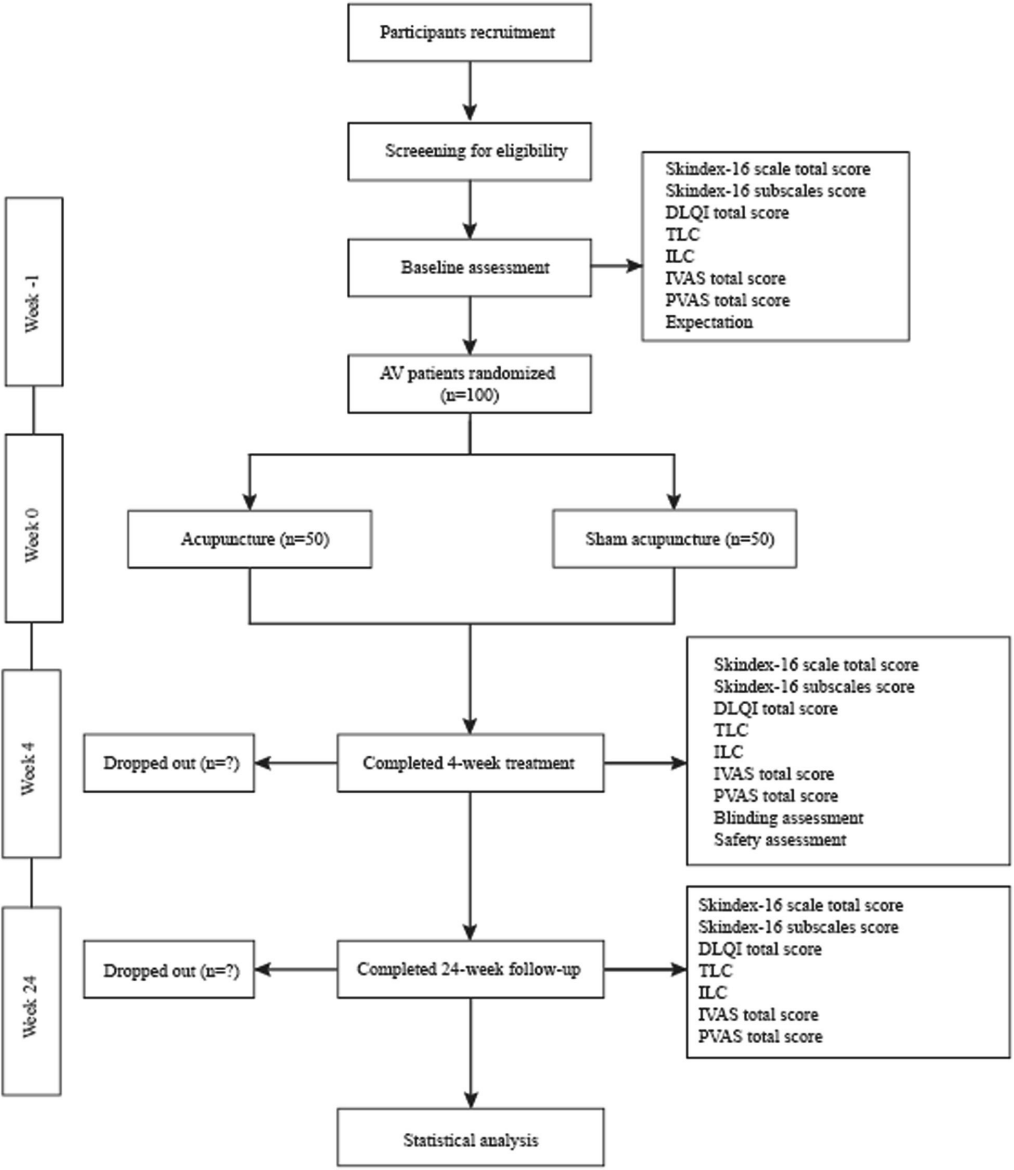
Schematic illustration of the study protocol

The participants, outcome assessors, and statisticians will be blinded to the group allocation. However, because of the characteristics of acupuncture, acupuncturists will not be blinded in this trial. To assess the blinding effect of sham acupuncture, participants will be asked to answer the following questions within 5 min after any treatment in week 4: “Do you think you have received traditional acupuncture?” The response options will be “yes,” “no,” or “unclear.” The study protocol is illustrated in Fig. 1.

### Ethical approval

This clinical trial will adhere to the principles of the Declaration of Helsinki and has been approved by the Ethics Committee of the Guang’anmen Hospital (2018-137-KY-01) (see Additional file 1).

### Participants

One hundred eligible participants with AV will be recruited in the trial.

### Inclusion criteria

1. Individuals aged between 18 and 48 years who qualify for the diagnostic criteria for AV [2, 24]
2. GAGS score between 19 and 38 [26]

### Exclusion criteria

Participants with any of the following conditions will be excluded:

1. Individuals with polymerization acne, explosive acne, drug acne, premenstrual acne, cosmetic acne, occupational acne, or any other subtypes of acne
2. Individuals with other severe diseases that may affect acne, such as polycystic ovary syndrome, thyroid disease, or atypical congenital adrenal hyperplasia
3. Individuals with other skin diseases that may influence the assessment of AV, such as rosacea, folliculitis, or other skin diseases
4. Individuals who have received antibiotics, retinoic acid, steroids, or anti-inflammatory drugs in the preceding 1 month
5. Individuals with severe heart, liver, kidney, hematopoietic system or autoimmune disorders, or severe systemic malnutrition
6. Pregnant and lactating women or those planning to conceive within 12 months
7. Individuals who have received acupuncture treatment in the past 3 months

### Intervention

All participants will receive treatment for 4 weeks with three sessions per week (ideally every other day), for a total of 12 sessions. Acupuncturists with an undergraduate degree or above and clinical experience of over 1 year at the Guang’anmen Hospital will be responsible for treatment. Participants will be treated separately to avoid communication during the trial period. We will discourage all included participants from receiving any other treatment for AV during the study period. If participants receive any other treatment for AV, they will also be included in this study, and all details of the other treatment for AV will be recorded in the case report form. We will compare the proportion of participants using other treatments between the two groups.

### Acupuncture group

The location of acupoints is described as per the Nomenclature and Location of Acupuncture Points (National Standard of People’s Republic of China, 2006 [GB/T 12346-2006]) [27]. The selection of acupoints will be decided with reference to the Guidelines for AV treatment in China (revised version 2014) [28]. Participants in the acupuncture group will receive stimulation at Dazhui (CV14), bilateral Quchi (LI11), bilateral Hegu (LI4), bilateral Zusanli (ST36), and bilateral Neiting (ST44) with disposable acupuncture needles (0.30 × 40 mm, Huatuo Brand; Suzhou Medical Appliance, Suzhou, Jiangsu, China). After routine disinfection, acupuncture needles will be inserted obliquely into CV14 to a depth of 30–40 mm at an angle of 15–30 degrees; acupuncture needles will be vertically inserted into bilateral LI11, bilateral LI4, bilateral ST36, and bilateral ST44 to a depth of 25–30 mm three times (once every 10 min) with slight lifting, thrusting, and twisting manipulations to produce a sensation of de-qi. Each session of acupuncture will last 30 min.

### Sham acupuncture group

Participants in sham acupuncture group will receive sham acupuncture at sham CV14 (10 mm to CV14), LI11 (10 mm to LI11), LI4 (10 mm to LI4), ST36 (25 mm to ST36), and ST44 (10 mm to ST44) with disposable acupuncture needles (0.30 × 25 mm, Huatuo Brand). Sham CV14, LI11, LI4, ST36, and ST44 will be vertically inserted to a depth of 1–2 mm without any manipulation and de-qi. The treatment sessions will last 30 min.

Participants will not be allowed use of other treatments for AV throughout the trial. Detailed information pertaining to use of other treatments will be recorded in the case report form.

### Rescue medication

Participants will not be encouraged to receive any other treatment or medication during the study period to prevent any influence on the results. However, in case of deterioration of the condition of AV during the 4-week treatment period and the 24-week follow-up, oral minocycline hydrochloride capsules will be prescribed to the patients (100 mg per day for 7 days) to relieve the symptoms of pain or itching. Details of the medication used will be recorded in the case report form. The proportion of participants using rescue drugs in the groups will be compared.

### Outcome measures

The primary outcome will be the change in the total score of the Skindex-16 scale from baseline at the end of 4-week treatment. The Skindex-16 scale is a brief, skin-related QoL scale with satisfactory reliability and validity [20, 21]. It is used to evaluate the efficacy of acupuncture in improving the QoL of patients with AV [20, 21]. The scale includes a total of 16 items that are categorized into three domains: the symptoms of participants with AV, the emotions of participants with AV, and function of participants with AV [20, 21]. The Skindex-16 scale score ranges from 0 (best) to 100 (worst), with a minimal clinically important difference (MCID) as 10 [20, 21].

### Secondary outcomes

1. The change from baseline in the Skindex-16 scale total score at weeks 16 and 28
2. The change from baseline in the Skindex-16 subscale (the symptoms of participants with AV, the emotions of participants with AV, and functioning of participants with AV) scores at weeks 4, 16, and 28
3. The change from baseline in the Dermatology Life Quality Index (DLQI) [29] scale total score at weeks 16 and 28. The DLQI scale is a tool used to assess the health-related QoL of patients with skin diseases. It has a total of ten items. The total score of DLQI ranges from 0 (best) to 30 (worst), with 4 as the MCID in inflammatory skin diseases [30].
4. The change from baseline in the total lesion (inflammatory and noninflammatory lesions) count (TLC) [31, 32] at weeks 4, 16, and 28
5. The change from baseline in the inflammatory lesion count (ILC) [26] at weeks 4, 16, and 28. Inflammatory lesions include inflammatory papules, pustules, and cysts. Noninflammatory lesions include blackhead and whitehead comedones. The lesions are assessed on the forehead, cheeks, nose, and chin. The inflammatory and noninflammatory lesions are counted from the pictures of the face obtained by a dermatologist using a digital camera.
6. The change from baseline in the degree of itch assessed using the Itch Assessment with Visual Analogue Scale (IVAS) [32] at weeks 4, 16, and 28
7. The change from baseline in the severity of pain assessed by visual analogue scale (PVAS) [33] at weeks 4, 16, and 28
8. The participants’ expectations of acupuncture will be assessed at baseline using the following two questions: “Do you think acupuncture will be effective for treating the illness?” and “Do you think acupuncture will be effective for relieving the related symptoms of AV?” The response options will be “yes,” “no,” or “unclear.”

The details of the evaluation of outcomes are shown in Table 1.

**Table 1:** Time points for assessment of outcomes

Outcome measures	Enrollment and allocation	Treatment				Follow-up	
	Baseline	Week 1	Week 2	Week 3	Week 4	Week 16	Week 28
Skindex-16 total score	X				X	X	X
Skindex-16 subscales score	X				X	X	X
DLQI total score	X				X	X	X
TLC	X				X	X	X
ILC	X				X	X	X
IVAS score	X				X	X	X
PVAS score	X				X	X	X
Blinding assessment					X		
Expectation	X						
Safety assessment		X	X	X	X		

*Abbreviations: DLQI* Dermatology Life Quality Index scale, *ILC* Inflammatory lesion count, *IVAS* Itch assessment with visual analogue scale, *PVAS* Pain assessment with visual analogue scale, *TLC* Total lesion count

### Safety evaluation

Adverse events (AEs), including AEs related to acupuncture (broken needle, local hematoma, infection, abscess, and others), AEs related to postacupuncture (nausea, vomiting, palpitations, dizziness, headache, insomnia, or any other symptoms after acupuncture treatment), and AEs unrelated to treatment will be recorded. For safety assessment, details of all AEs will be recorded by a research assistant in the case report form. Any serious adverse events will be reported within 24 h to the Ethics Approval Committee of the Guang’anmen Hospital of China Academy of Chinese Medical Sciences. The Department of Acupuncture of the Guang’anmen Hospital of China will provide insurance coverage to compensate for any injuries related to the interventions during this study.

### Sample size and statistical analysis

The sample size of this trial was calculated on the basis of primary outcome, which is the change from baseline in the Skindex-16 scale score at the end of week 4. In our unpublished pilot trial, the mean (± standard deviation) reductions in the Skindex-16 scale score after 4-week treatment in the acupuncture and sham acupuncture groups were 12.50 ± 19.09 and 0.40 ± 21.12, respectively. Assuming an alpha risk of 5% and a beta risk of 20%, a sample size of 100 (50 participants in each group) was calculated, considering a 20% dropout rate.

The data will be analyzed using IBM SPSS Statistics version 20.0 software (IBM Corp, Armonk, NY, USA) according to the intention-to-treat principle. Normally distributed continuous variables will be reported as mean ± standard deviation or 95% confidence intervals; non-normally distributed continuous variables will be reported as median (interquartile range). Categorical variables will be presented as frequency (percent). For data pertaining to dropouts, the actual observational value of the last observation will be used for statistical analysis. Between-group differences with respect to normally distributed continuous variables will be assessed using analysis of variance, and those with respect to non-normally distributed continuous variables will be assessed using nonparametric tests for the primary outcome and the secondary outcomes. Data pertaining to the participants’ expectations of acupuncture and the safety evaluation will be analyzed using the chi-square test or Fisher’s exact test. For the blinding assessment, the percentage of participants choosing traditional acupuncture will be assessed using the chi-square test. All *P* values will be two-tailed; *P* < 0.05 will be considered indicative of statistical significance.

### Quality control

All researchers will take a training course before the beginning of this trial. Due measures will be implemented to ensure the traceability and confidentiality of the case report form, informed consent form, and other original data. A double-input method will be used for data entry. AEs will be recorded in detail, properly handled, and tracked. All trial-related procedures and data management will be supervised. The Data Monitoring Committee of the Guang’anmen Hospital will regularly monitor the recruitment and screening of participants, data collection, monitoring, and verification of AEs to ensure that the study is conducted in accordance with the approved protocol.

## Discussion

AV is a common chronic inflammatory dermatologic disease. It may affect the physical and mental health of patients and may adversely affect their QoL [34]. According to an epidemiological study, approximately 20% of young people are affected by moderate to severe AV [35]. Several studies have demonstrated a correlation between the QoL and the severity of dermatologic disease [36]. For this reason, this study will only include participants with moderate or severe AV.

The primary outcome of the study will be the Skindex-16 scale score. It is a validated and sensitive scale to measure the QoL of patients with dermatologic diseases [20, 21]. Because lesions of AV mainly occur on areas of the face and are accompanied by other symptoms of itch and pain [32, 33], patients tend to present with psychosocial symptoms and impaired QoL. The Skindex-16 scale consists of 16 questions in the symptoms, emotion, and function domains; responses to each question are scored on a 7-point Likert scale [20, 21]. This scale includes seven questions related to the symptoms (itch or pain) and characteristics (lesions) of AV. It is a sensitive and specific tool to assess the symptoms and QoL of patients with moderate or severe AV. Moreover, we also used the DLQI scale to evaluate QoL. It is a brief, skin-related QoL scale with satisfactory reliability and validity, and it was used to further evaluate the effectiveness of acupuncture in improving the QoL of patients with AV [29]. In addition, TLC, ILC, IVAS, and PVAS will also be used as secondary outcomes to assess the lesion counts and main symptoms (itch or pain) related to AV. These would provide a comprehensive evaluation of the overall effectiveness of acupuncture.

In this trial, we aim to evaluate the therapeutic effect of acupuncture on the symptoms and QoL of patients with moderate or severe AV. The possible placebo effects of acupuncture may be attributable to the participants’ expectations of acupuncture; the degree of trust between the acupuncturist and the participant; and any difference in the type, frequency, or course of the intervention of acupuncture [37, 38]. To partially exclude the placebo effects and demonstrate the real effect of acupuncture, we plan to use sham acupuncture as a comparator.

The sham acupuncture is a crucial tool that can minimize participants’ expectations and the bias of both participants and researchers and assist in the enforcement of blinding [38]. Use of the non-insertion-type needle on nonacupoints was shown to be an optimal method to reduce the possible biological effects [39]. However, the non-insertion-type needle leaves no marks or wounds on the skin. In this condition, the lack of any needle marks may compromise the blinding of participants. Therefore, we will opt for nonacupoints and minimal acupuncture as the sham acupuncture in this trial. Nonacupoints are in proximity to the classical acupoints, which will increase the feasibility of blinding [38]. Meanwhile, using the nonacupoints and minimal acupuncture without any manipulation may minimize actual needle sensation to mitigate the potential physiologic stimulus [38, 40]. The outcome assessors of this study will be blinded to the group allocation, which will also mitigate the lack of acupuncturist blinding.

However, some limitations of our study should be acknowledged. First, the single-center scope of the study and the relatively small sample size may lead to overestimation of the effects of acupuncture. Second, because of the characteristics of acupuncture, the acupuncturist will not be blinded in our trial, which may cause potential bias. Last, it is difficult for the sham acupuncture to be as inert as desired in this trial, owing to a slight stimulus or a stronger stimulus of gentle touch possibly leading to different degrees of neurological responses related to the treatment effect [39, 41]. So, the use of nonacupoints and minimal acupuncture without any manipulation may cause some biological effect leading to false-negative results [36].

### Trial status

No recruitment currently.

## Supplementary information

**Additional file 1.** Ethics approval of Guang’anmen Hospital of China Academy of Chinese Medical Sciences.

## Data Availability

All relevant data will be shared for a period beginning 3 months after publication and ending 5 years after publication.

## Abbreviations

AE: Adverse event
AV: Acne vulgaris
DLQI: Dermatology Life Quality Index
GAGS: Global Acne Grading System
ILC: Inflammatory lesion count
IVAS: Itch Assessment with Visual Analogue Scale
MCID: Minimal clinically important difference
PVAS: Pain Assessment with Visual Analogue Scale
QoL: Quality of life
SPIRIT: Standard Protocol Items: Recommendations for Interventional Trials
STRICTA: Standards for Reporting Interventions in Clinical Trials of Acupuncture
TLC: Total lesion count
