# Supplementary material for: Efficacy of acupuncture in improving the symptoms and the quality of life of patients with moderate or severe acne vulgaris: study protocol for a randomized controlled trial
Source: Trials. 2020 Jun 23;21:563. doi: 10.1186/s13063-020-04346-7 (PMC7310417; doi:10.1186/s13063-020-04346-7)
Supplement: Supplementary file 1 — Additional file 1. Ethics approval of Guang’anmen Hospital of China Academy of Chinese Medical Sciences. [file 13063_2020_4346_MOESM1_ESM.pdf]

# Ethics Approval of Guang’anmen Hospital of China Academy of Chinese Medical Sciences

| | | | |
|---|---|---|---|
| Project Title | Efficacy of acupuncture in improving the quality of life of patient’s with moderate or severe acne vulgaris: protocol for a randomized controlled trial | | |
| Approval No. | 2018-137-KY-01 | Project Sponsor | Investigator |
| Research Organization | Guang’anmen Hospital of China Academy of Chinese Medical Sciences | | |
| Applicant | None | | |
| Site PI | Zliishun Liu | | |
| Review Attribute | Initial Review | Review Methods | Review Conference |
| Review Date | December 20,2018 | Review Place | Conference Room, the 4th Floor of the Administrative Building of Guang’anmen Hospital |
| Review Committee | Haibo Yin, Lan Lin, Xinghua Feng, Limin Xie, Ling Feng, Ping Wu, Lizhen Gu, Mei Han | | |
| Approval Files | 1. Study Protocol (Version No. 2018120301; Version Date: Dec 03,2018)<br>2. Case Report Form (Version No. 2018120301; Version Date: Dec 03,2018)<br>3. Informed Consent (Version No. 2018120301; Version Date: Dec 03,2018)<br>4. Recruiting Advertisement (Version No. 2018120301; Version Date: Dec 03,2018)<br>5. Researchers’ Handbook Version No. 2018120301; Version Date: Dec 03,2018) | | |
| Review Comments | According to “ethical review methods for biomedical study involving humai subjects” issued by the Ministry of Health, “Good Clinical Practice’’, “Provisions for Clinical Trials of Medical Device” and “Guidelines for Ethical Review Work of Drug Clinical Trials” issued by State Food and Drug Administratio (SFDA) of the People’s Republic of China, "management specifications for ethica review of TCM clinical studies” issued by State Administration of Traditional Chinese Medicine, “Declaration of Helsinki’’, and “ International ethical guidelines for biomedical research involving human subjects” issued by Council fo International Organizations of Medical Sciences, this clinical research was reviewe by the institutional review board (IRB) of Guang'anmen Hospital of China Academ of Chinese Medical Sciences. And the study protocol, informed consent, and th recruitment files of this research were approved.<br>Please conduct this clinical study following the GCP principles and the study | | |

| | |
|---|---|
| | protected throughout the whole study.<br>The ethical approval will invalid if the study didn't carry out within 3 years. Th<br>researchers are required to apply for ethical review once again.<br>An application should be submitted if a change of the principle investigator (PI), o<br>any modification of the study protocol, informed consent, or the<br>recruitment files are made.<br>A report of the severe adverse events (SAE) should be submitted within 15 days i<br>any SAE or any other un-anticipated AE, which will affect the risk- reward ratio o<br>this study, occurs. A report should be submitted immediately if lethal adverse event<br>are aware of.<br>Researchers should submit report of the study progress one month before th<br>deadline according to ethical review frequency. A summary report of the stud<br>progress of each site should be submitted by the site PI to the IRB of the leading sit<br>In any condition which will greatly affect the progress of the study or increase th<br>potential risk of the subjects, a written report should be submitted by the site PI to th<br>IRB.<br>A protocol deviation report should be submitted by the site<br>P I/mon itor/researc her if any of the following occurs: 1) conditions that violate th<br>study protocol: subjects who did not meet the inclusion criteria, or should b<br>excluded according to the exclusion criteria, were wrongly included in the study<br>subjects do not withdraw from the study when he/she meet the rules of withdrawal<br>incorrect treatment or dose was given; prohibited combined medicine was used; 2<br>conditions that violate GCP principle: subjects' rights and health are badly affected<br>the science of study was badly affected.<br>A final report should be submitted when the study is finished completely or<br>terminated prematurely. |
| Validity Period of the Approval | From Dec 25,2018 to Dec 24,2019 |
| Frequency of Follow-up Review | 12 months Deadline of Follow-up Review Dec 24,2019 |
| Contact | Contact person: Jie Qiao Contact telephone: +86 01088001552 E-mail: gamhec@126.com |
| Director/Vice Director Signature | Haibo Yin |

IRB of Guang'anmen Hospital of China Academy of Chinese Medical Sciences (Seal)
Date: Dec 24, 2018
